# School-Based Health Centers and School Attendance in Rural Areas

**DOI:** 10.1001/jamanetworkopen.2025.10083

**Published:** 2025-05-13

**Authors:** Chris Kjolhede, Wendy M. Brunner, John W. Sipple

**Affiliations:** 1 Bassett Healthcare Network, Cooperstown, New York; 2 Research Institute, Bassett Medical Center, Cooperstown, New York; 3 Department of Global Development, Cornell University, Ithaca, New York

## Abstract

**Importance:**

School-based health centers (SBHCs) provide students with convenient access to physical, mental, and dental health services, which is particularly important in rural areas with long travel distances and limited availability of primary care.

**Objective:**

To examine the association between SBHCs and school attendance in a rural region.

**Design, Setting, and Participants:**

This cross-sectional study compared attendance rates among students in school districts with and without SBHCs in rural upstate New York. Student attendance data from the 2015 to 2016 through the 2018 to 2019 school years were obtained for 52 schools in 32 districts within a regional education service area. Analysis included students in kindergarten through 12th grade from 18 schools in 14 districts with SBHCs and 34 schools in 18 districts without SBHCs. Multivariable logistic regression was used to model the association between SBHC access and risk of chronic absenteeism. Statistical analysis was performed from May 2024 to February 2025.

**Exposure:**

Access vs no access to an SBHC based on which district a student was enrolled in. In districts with SBHCs, all schools had SBHCs.

**Main Outcomes and Measures:**

Absenteeism was calculated as the number of days absent divided by the total days enrolled and classified by federal and state chronic absenteeism categories: not at risk (0%-4.99% absent), at risk of chronic absenteeism (5%-9.99% absent), and chronically absent (≥10% absent).

**Results:**

Attendance data were available for 66 303 students (kindergarten through 12th grade; 49.4% female) during 4 years: 30 046 from SBHC districts and 36 257 from non-SBHC districts. Across all but 1 school year, non-SBHC students were significantly more likely than SBHC students to be classified as chronically absent or at risk for chronic absenteeism. Students in SBHC districts had 12% greater odds of being not at risk for chronic absenteeism after accounting for grade, sex, school year, economic disadvantage, and community characteristics of wealth and district size (odds ratio, 1.12; 95% CI, 1.08-1.16). Evidence of a stronger association was found between SBHC access and reduced absenteeism among elementary school students and among children attending schools with higher student poverty and higher community wealth.

**Conclusions and Relevance:**

This cross-sectional study of rural students in kindergarten through 12th grade found that students in SBHC districts had significantly fewer absences than students in non-SBHC districts in the same region. These findings suggest that by providing primary care services at school, SBHCs may help decrease absenteeism among students in rural communities.

## Key Points

**Question:**

Is there an association between school-based health centers (SBHCs) and student absenteeism in rural areas?

**Findings:**

In this cross-sectional study of 66 303 students in kindergarten through 12th grade, those with access to SBHCs had significantly fewer absences than students with no access to SBHCs in the same rural region after accounting for grade, sex, school year, economic disadvantage, community wealth, and school district size. These differences were greatest at lower levels of absenteeism and among elementary students.

**Meaning:**

These results suggest that by providing primary care services at school, SBHCs may decrease absenteeism among students in rural communities.

## Teaser

This cross-sectional study assesses whether student absenteeism is less frequent in rural school districts with vs without school-based health centers.

## Introduction

Children living in rural areas of the US are more likely to experience barriers to accessing health care due to long travel distances, a lack of public transportation, and limited availability of health care professionals.^1,2,3^ Parents may need to take their children long distances for medical appointments, leading to more missed school and missed work time (potentially including lost wages) for parents.

School-based health centers (SBHCs) are clinics that provide an accessible source of physical, mental, and dental health services to students on school campuses.^4^ The services that SBHCs typically provide include annual well-child visits, immunizations, acute care, chronic disease management, mental health care, and oral health care. Rural students are more likely to use SBHCs.^5,6,7^ There are currently an estimated 3900 SBHCs in 48 states, the District of Columbia, and Puerto Rico.^8^

Chronic absenteeism is associated with poverty and is as likely to be prevalent in rural compared with urban and suburban areas.^9,10^ In 2023, the average daily attendance in public schools in the US was 90%, with 15% of schools extremely concerned about student absenteeism.^11^ As documented in a policy statement from the American Academy of Pediatrics, chronic absenteeism is associated with poor health outcomes.^12^

Previous studies have demonstrated mixed findings related to SBHCs and absenteeism. A 2004 review of SBHCs and academic outcomes^13^ reported that 3 of the 6 studies reviewed that had measured attendance had documented a positive association between use of SBHCs and school attendance,^14,15,16^ whereas 3 other studies did not.^17^ A subsequent controlled, longitudinal comparison of users and nonusers of SBHCs in urban Seattle showed that use of SBHC medical services was associated with a significant increase in school attendance.^18^ A 2023 study^19^ in a large urban school district examined the change in school attendance before and after using an SBHC, comparing SBHC users with nonusers. The authors found decreases in absenteeism associated with SBHC use, with the biggest differences among students with a mental health diagnosis. Finally, a recent study^20^ of students in kindergarten through eighth grade attending urban schools demonstrated a greater decrease in absenteeism among SBHC enrollees compared with students not enrolled in an SBHC.

Fewer studies have assessed the association of SBHCs with school attendance in rural communities. Our study examined the association of SBHCs with school attendance in school districts with SBHCs compared with districts in the same rural region without SBHCs. We hypothesized that student absenteeism would be less frequent in a school district with vs without an SBHC.

## Methods

### Setting

This cross-sectional study examined the association between attendance and SBHC exposure in the 2015 to 2016 through the 2018 to 2019 school years among students attending schools in a 4-county region in upstate New York where a health care system operates a network of SBHCs. Student race and ethnicity were not included with the attendance data provided to us. The Cornell University institutional review board exempted this study from review because it was a secondary data analysis of deidentified data; therefore, informed consent was not required. We followed the Strengthening the Reporting of Observational Studies in Epidemiology (STROBE) reporting guideline.

The 4-county region is rural, with whole counties designated as health professional shortage areas for general medical, mental, and dental health care. Public transportation is scarce, and measures of socioeconomic development fall below state averages.^21,22^ School districts are small and typically have all students in 1 (kindergarten through 12th grade) or 2 (elementary and middle plus high school) buildings, often on the same campus. Among the 14 districts with SBHCs included in this analysis, 3 have 2 SBHCs, 1 for elementary and 1 for middle plus high school. All districts are independently managed by local boards of education and served by 1 of 3 regional boards of cooperative education services. Comparison districts (those without SBHCs) are similarly situated and often geographically adjacent to SBHC districts. The SBHCs offer comprehensive medical, dental, and mental health care to all students in these school districts.

SBHCs sponsored by this health care system are part of the system’s department of pediatrics. Each SBHC has a supervising pediatrician. There is one model of care, and all sites conform to state department of health guidelines, American Academy of Pediatrics policies, and Centers for Disease Control and Prevention recommendations. Most are recognized as patient-centered medical homes. Schools initiate the process of opening an SBHC, typically based on community desire to improve access to care for students. As of 2015, all but 1 SBHC had been open for 3 or more years, with the oldest being open for 23 years. The mean duration of operation of these SBHCs was 10 years. Among students attending schools with one of these SBHCs, the mean rate of enrollment in the SBHCs was greater than 80%.

### Data Sources

The data for this study came from 2 sources. First, we obtained demographic, fiscal, and performance variables from New York State via NYEducationData.org. Second, through a data use agreement with a board of cooperative education services, we obtained individual deidentified student attendance data from the New York State Information Reporting Service compiled by a regional information center, including student-level data from 52 schools in 32 districts (18 schools in 14 districts with SBHCs and 34 schools in 18 districts without SBHCs) from school year 2015 to 2016 through school year 2018 to 2019. Attendance data were coded before analysis according to state reporting requirements as absent (in any form, excused or unexcused), present (including tardy or late), or unknown. We include only data beginning with the 2015 to 2016 school year because that is the beginning of the availability of standardized attendance data (per Every Student Succeeds Act [ESSA] rules) and before the 2019 to 2020 academic year because the COVID-19 pandemic disrupted both attendance and data collection. We then linked district data and attendance data (via a unique district identification number) with the list of all districts in the region served by the health care system and identified which districts had SBHCs. In districts with SBHCs, all schools had SBHCs.

We measured absenteeism as the number of school days absent divided by the total number of days enrolled in school. Descriptive and bivariate analyses were conducted with the continuous version of this attendance variable. We classified the absence percentage using the New York State Education Department (NYSED) attendance categories (based on federal guidance in ESSA 2015): not at risk (0%-4.99% absent), at risk of chronic absenteeism (5%-9.99% absent), and chronically absent (≥10% absent). This categorization allowed for policy-relevant effects and resulted in primary use of a dichotomous dependent variable with a value of 0 for at risk or chronically absent and a value of 1 for no risk of chronic absenteeism.

SBHC status (access vs no access to an SBHC) is indicated by whether a student attended school in a district with or without an SBHC. We included all students attending SBHC districts in the analysis whether or not they were enrolled to receive care from the SBHC, because all were exposed to the presence of an SBHC. We excluded prekindergarten students (n = 2736) because not all schools had a prekindergarten program, students enrolled in a fifth or sixth year of high school (n = 352), and kindergarten through 12th-grade students enrolled in school fewer than 130 school days (n = 5735) because we determined these students would not have had sufficient time of exposure to the SBHC. Additionally, we excluded students absent for greater than 30% of total enrolled days (n = 393) because this could be an indication of issues not impacted by school-based health (eg, moving between districts, withdrawing from school, or major illness). These last 2 attendance-based rules excluded 8.46% of students and resulted in a final sample of 66 303 students in kindergarten through 12th grade. Analyses of variance found no significant differences in absenteeism risk by SBHC status among the excluded observations.

We used school district information to demonstrate that the SBHC and non-SBHC districts being compared were similar by socioeconomic status and district size. We used the percentage of students determined to be economically disadvantaged (common federal and state definition after the passage of ESSA)^23^ obtained from NYSED as one indicator of socioeconomic status. We also used a broader measure of community wealth, combined wealth ratio, a combination of average total taxable property wealth of the community per pupil and average household income across the community per pupil converted into deciles, with the lowest decile representing the poorest communities or districts and the highest decile indicating the wealthiest.^24^ Finally, we controlled for the size of the communities by using the total kindergarten to 12th grade enrollment.

### Statistical Analysis

We summarized the distribution of percentage of days absent per district per school year by SBHC status using means (SDs) and medians (IQRs). Because the distribution of proportion of days absent was right skewed, we used the nonparametric Wilcoxon rank sum test to compare median absence percentage by SBHC status. We used χ^2^ tests to detect differences in absence percentage by categories of chronic absenteeism. We used multivariable logistic regression to model the association between SBHC status and the odds of being in the not at-risk category of absence (0%-4.99%), accounting for school year, grade, sex, district size, economic disadvantage, and community wealth. We selected this outcome variable because it allowed us to examine students moving from chronically absent or at risk of chronic absence to the not at risk of chronic absenteeism category because that is where we expected to see an association. We included selected interaction terms in the final model to assess the unique association of the SBHC with individual and community-level characteristics. We tested for a statistically significant cluster effect of students attending schools within districts in the unconditional model, finding that less than 5% of the variability in attendance was accounted for by district in which students were enrolled. Thus, we did not run multilevel models for the 66 303 observations across the 4 years of data. Finally, in a sensitivity analysis, we ran the logistic models separately by school year to check for differences in the SBHC-absenteeism association. Statistical analysis was performed from May 2024 to February 2025. A 2-sided *P* < .05 was considered statistically significant. All statistical analyses were conducted using Stata software, version 18 (StataCorp LLC).

## Results

Attendance data were available for 66 303 students in kindergarten through 12th grade during 4 years: 30 046 from 18 schools in 14 SBHC districts and 36 257 from 34 schools in 18 non-SBHC districts. Overall, 49.4% of students were female. There were no statistically significant differences in school district characteristics by SBHC status. Non-SBHC districts had slightly higher mean (SD) community wealth deciles compared with SBHC districts (6.9 [2.5] vs 5.0 (3.1]; *P* = .07) and similar proportions of students experiencing economic disadvantage (Table 1).

**Table 1.** Characteristics of Students and School Districts in Districts With and Without SBHCs, 2018-2019

Characteristic	No SBHC (n = 18 districts [34 schools])		SBHC (n = 14 districts [18 schools])		*P* value
	Mean (SD)	Median (IQR)	Mean (SD)	Median (IQR)	
K-12 district enrollment	539.1 (474.3)	329.5 (254.0-754.0)	558.0 (338.1)	356.0 (327.0-770.0)	.90
No. of days enrolled	177.5 (8.7)	174.9 (173.5-177.9)	175.9 (3.0)	175.9 (172.9-177.3)	.46
No. of days present	168.9 (8.7)	167.0 (165.1-169.2)	167.8 (3.3)	167.4 (164.1-169.2)	.48
No. of days absent	8.6 (1.8)	8.5 (7.7-10.2)	8.1 (2.5)	8.1 (7.1-9.4)	.90
Days absent, %	4.9 (1.0)	4.8 (4.4-5.7)	4.6 (1.4)	4.7 (4.1-5.3)	.96
No risk for chronic absence, %	60.1 (9.7)	61.1 (51.4-66.3)	64.3 (13.6)	62.8 (56.5-69.1)	.68
At risk for chronic absence, %	28.4 (5.5)	28.8 (25.8-32.8)	24.9 (7.1)	26.1 (23.4-29.0)	.24
Chronically absent, %	11.5 (5.5)	10.9 (8.2-14.6)	10.8 (7.6)	10.1 (7.5-15.0)	.74
Sex, %					
Male	50.2 (2.2)	50.8 (48.2-51.4)	50.5 (2.6)	50.9 (48.4-52.2)	.73
Female	49.8 (2.2)	49.2 (48.6-51.8)	49.2 (2.9)	49.1 (47.8-51.6)	
School, %					
Elementary	44.5 (3.3)	45.5 (41.8-46.1)	48.9 (10.8)	46.0 (44.2-47.0)	.17
Middle	23.7 (2.3)	24.2 (21.7-25.7)	23.0 (3.1)	23.4 (22.7-24.9)	.92
High	31.8 (3.3)	31.3 (30.3-32.2)	28.1 (8.3)	30.1 (29.4-32.0)	.20
Community wealth ratio	1.06 (0.79)	0.73 (0.55-1.25)	0.67 (0.25)	0.57 (0.49-0.74)	.08
Community wealth decile	6.9 (2.5)	7.0 (5.0-9.0)	5.0 (3.1)	5.0 (2.0-7.0)	.07
Economically disadvantaged, %	56.5 (9.0)	57.4 (55.4-58.6)	53.3 (11.9)	56.8 (51.5-59.7)	.41
School tax rate per $1000, $	16.00 (5.80)	15.22 (12.75-18.39)	17.36 (4.41)	17.99 (14.13-20.58)	.47
Total expenditure per pupil, $	30 977 (7205)	28 637 (26 215-34 938)	27 782 (3663)	27 595 (24 528-31 309)	.14

Abbreviations: K-12, kindergarten to 12th grade; SBHC, school-based health center.

For the total sample, mean (SD) absence was 4.9% (1.1%) of enrolled school days and median (IQR) absence was 4.7% (4.3%-5.5%). The total range of absence was 0% to 21% of enrolled days. Median (IQR) absence was 4.8% (4.4%-5.7%) in non-SBHC districts compared with 4.7% (4.1%-5.3%) in SBHC districts in 2018 to 2019 (*P* = .96). Maximum absence in 2018 to 2019 in non-SBHC districts was 6.8% compared with 7.9% in SBHC districts. The distribution of absence percentage skewed higher among students in non-SBHC districts compared with SBHC districts (Figure) in each school year, with the biggest differences by SBHC status observed in the range of 10% or less enrolled days absent.

**Figure.**
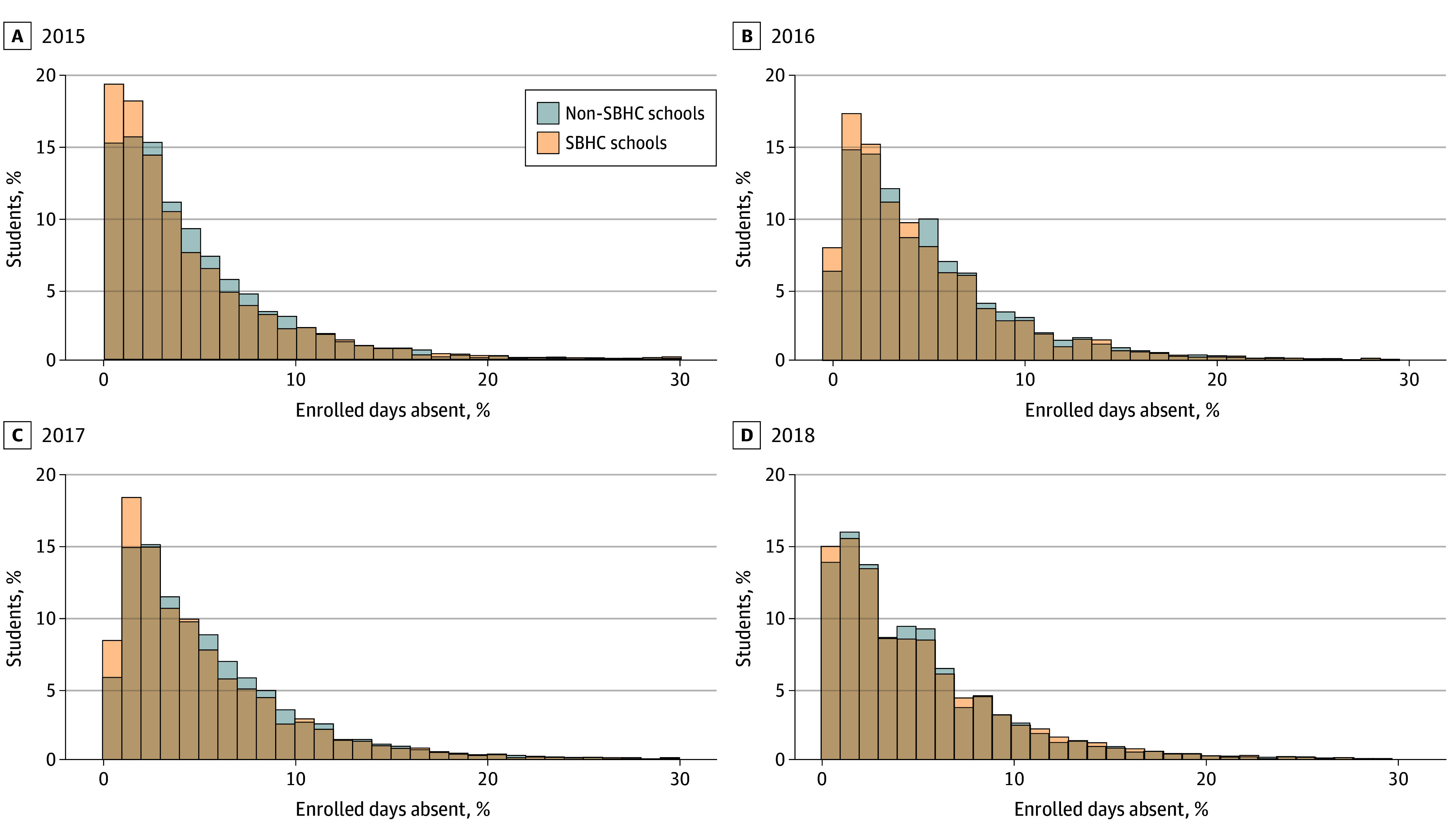
Distribution of Percentage of Enrolled Days Absent for Students in School Districts With and Without a School-Based Health Center (SBHC) by Year

By absenteeism category, 5131 students (69.5%) in SBHC districts met the not-at-risk criteria of less than 5% days absent compared with 5946 students (66.1%) in the non-SBHC districts in 2015 to 2016 (Table 2). These differences held across 2016 to 2017 and 2017 to 2018 with 5.0– and 5.3–percentage point advantages, respectively. In 2018 to 2019, non-SBHC districts had a higher percentage at no risk; however, the distribution by risk category was not statistically significant (χ^2^ = 5.90; *P* = .052) in that school year. Students in the non-SBHC districts were more likely to be in the at-risk category or chronically absent categories (with the exception of 2015 to 2016 for chronic absenteeism, although the percentage point difference was 0.2). The proportion of students who were at risk was 3.7, 3.9, 4.6, and 0.6 percentage points greater in 2015 to 2016, 2016 to 2017, 2017 to 2018, and 2018 to 2019, respectively, for those in non-SBHC districts.

**Table 2.** Distribution of Students by Chronic Absenteeism Categories in Schools With and Without SBHCs

Category	2015-2016				2016-2017				2017-2018					2018-2019			
	No SBHC, No. (%) (n = 8997)	SBHC, No. (%) (n = 7383)	χ^2^	*P* value	No SBHC, No. (%) (n = 9088)	SBHC, No. (%) (n = 7626)	χ^2^	*P v*alue		No SBHC, No. (%) (n = 9194)	SBHC, No. (%) (n = 7541)	χ^2^	*P* value	No SBHC, No. (%) (n = 8979)	SBHC, No. (%) (n = 7496)	χ^2^	*P* value
Not at risk for chronic absenteeism (0%-4.99%)	5946 (66.1)	5131 (69.5)	31.25	<.001	5098 (56.1)	4658 (61.1)	43.31	<.001		5213 (56.7)	4676 (62.0)	52.77	<.001	5499 (61.3)	4548 (60.7)	5.90	.052
At risk for chronic absenteeism (5%-9.99%)	2137 (23.8)	1486 (20.1)			2760 (30.4)	2021 (26.5)				2728 (29.7)	1893 (251)			2416 (26.9)	1968 (26.3)		
Chronically absent (≥10%)	914 (10.2)	766 (10.4)			1230 (13.5)	947 (12.4)				1253 (13.6)	972 (12.9)			1063 (11.8)	980 (13.1)		

Abbreviation: SBHC, school-based health center.

Finally, multivariable logistic regression analyses confirm reduced absenteeism for students above and beyond student and district (eg, community) characteristics (Table 3). Model 1 shows that students in SBHC districts had 1.15 times greater odds of being in the not-at-risk category when compared with students in the non-SBHC districts after controlling for any change during the 4 years of data (odds ratio [OR], 1.15; 95% CI, 1.11-1.19). After accounting for year, sex, and poverty (model 2), there was no change in the association with SBHC status (OR, 1.15; 95% CI, 1.12-1.19). With the addition of community size and grade level (model 3), the association with SBHCs persisted (OR, 1.12; 95% CI, 1.08-1.16). The final model added in a set of interaction terms that had a statistically significant association when entered individually to assess the unique association of SBHCs to the individual- and community-level characteristics. We found that SBHC districts with 1 higher decile of poverty or 1 higher decile of community wealth each had 5% greater odds of membership in the not-at-risk group. We found a substantial increase in the odds of being not at risk for chronic absence among elementary school students with access to an SBHC compared with elementary school students without access. We found no unique association for high schoolers with access to an SBHC but continued to see the negative association of high school with attendance in general. Finally, our sensitivity analysis showed a statistically significant association of SBHCs with school attendance in 2015 to 2016, 2016 to 2017, and 2017 to 2018 but not in 2018 to 2019 (OR, 0.99; 95% CI, 0.93-1.06; *P* = .84)

**Table 3.** Logistic Regression Models of No Risk for Chronic Absenteeism by Student and Community Characteristics and Select Interaction Terms

Variable	Model 1		Model 2		Model 3		Model 4	
	OR (95% CI)	*P* value	OR (95% CI)	*P* value	OR (95% CI)	*P* value	OR (95% CI)	*P* value
SBHC	1.15 (1.11-1.19)	<.001	1.15 (1.12-1.19)	<.001	1.12 (1.08-1.16)	<.001	1.00 (0.96-1.05)	.96
Year^a^	0.92 (0.91-0.94)	<.001	0.92 (0.91-0.93)	<.001	0.92 (0.91-0.93)	<.001	0.92 (0.91-0.93)	<.001
Female	NA	NA	0.96 (0.93-0.99)	.01	0.96 (0.93-0.99)	.02	0.97 (0.93-0.99)	.02
Economic disadvantage decile^a^	NA	NA	1.01 (1.01-1.02)	<.001	1.00 (1.00-1.01)	.42	0.99 (0.98-1.00)	.04
Community wealth decile^a^	NA	NA	NA	NA	0.98 (0.97-.99)	<.001	0.97 (0.96-0.97)	<.001
K-12 enrollment^a^	NA	NA	NA	NA	1.00 (1.00-1.00)	.16	1.00 (1.00-1.00)	.01
Elementary grades	NA	NA	NA	NA	1.01 (0.97-1.05)	.61	0.94 (0.89-0.98)	.01
High school grades	NA	NA	NA	NA	0.75 (0.72-0.78)	<.001	0.75 (0.72-0.78)	<.001
SBHC × economic disadvantage	NA	NA	NA	NA	NA	NA	1.05 (1.04-1.06)	<.001
SBHC × community wealth	NA	NA	NA	NA	NA	NA	1.05 (1.04-1.07)	<.001
SBHC × elementary	NA	NA	NA	NA	NA	NA	1.21 (1.11-1.27)	<.001
Constant^b^	1.44 (1.41-1.47)	<.001	1.46 (1.42-1.50)	<.001	1.63 (1.57-1.70)	<.001	1.73 (1.66-1.80)	<.001

Abbreviations: K-12, Kindergarten through 12th grade; NA, not applicable; OR, odds ratio; SBHC, school-based health center.

^a^ Centered variable.

^b^ Constant represents the value of the dependent variable when all covariates are set to zero.

## Discussion

To our knowledge, this is the first study to demonstrate an association between rural SBHCs and school attendance. Overall, we found that students in districts with SBHCs had lower rates of absenteeism than their peers in districts without SBHCs. Multivariable logistic regression supported the association between SBHCs and reduced absenteeism above and beyond grade, sex, school year, economic disadvantage, and community characteristics of wealth and district size and showed a distinct association among elementary school children.

We suspect that students accessing SBHCs had improved attendance because of the easy availability of care. In the model we studied, the SBHCs were located on-site, allowing students to go directly from class to a medical appointment and then back to class, whereas an appointment with an outside clinician requires leaving school, resulting in missed class time and potentially an excused absence. Additionally, the presence of an SBHC could result in improved attendance by providing health care at no out-of-pocket cost to students who would not otherwise have access to care, leading to better health outcomes. The greater association among elementary students may be related to the fact that the infectious disease burden is higher and therefore the need for primary care is greater in that age group. Having an on-site source for health care obviates the need to leave school or not go to school (be absent).

We observed the greatest magnitude of difference in school absence between SBHC and non-SBHC districts at the lower levels of absenteeism (in the no-risk and at-risk categories), with little to no difference among students meeting the definition of chronic absenteeism. SBHCs address many of the individual risk factors linked to chronic absenteeism, including chronic illness, lack of access to medical or dental care, and mental health conditions.^25^ However, many of the risk factors for chronic absenteeism, such as housing instability, lack of supervision at home, and unreliable school transportation,^26^ may exist beyond the reach of SBHC care.

We found that students in SBHC districts with greater rates of student economic disadvantage were more likely than those in non-SBHC districts to be not at risk of chronic absenteeism, above and beyond the other independent variables (including community wealth, as indicated by property values and household income). However, students attending SBHC schools were also more likely to be not at risk of chronic absenteeism if community wealth was higher. The correlation between economic disadvantage and the community wealth ratio was only −0.37, which is negative but not as strong as might be expected. In this case, SBHC districts seem to better serve students from both higher rates of economic disadvantage (student measure) and in communities with higher property and/or income wealth (community measure).

The mechanisms linking SBHCs and lower school absenteeism rates have yet to be delineated. Indeed, the 2016 Community Guide Systematic Review of the role of SBHCs in addressing health equity did not include studies of SBHCs and school attendance due to a lack of “plausible or clear mechanisms of impact.”^26^ The reviewers note it is possible that the presence of an SBHC could lead to increases in school attendance if it leads parents to send their sick children to school to be treated or decrease school attendance if it leads to increased diagnoses of illness.^27^

School connectedness (a student’s sense of connection with schools and school personnel^27^) has been suggested as a factor linking SBHC use with improved academic outcomes.^13^ Strolin-Goltzman et al^28^ reported data from 3 urban schools showing that SBHC users had higher degrees of school connectedness than nonusers. Although they found significant differences in grade promotion and tardiness by SBHC status, they did not find a significant difference in attendance.

### Limitations

This study has limitations. Differences in attendance rates could be due to factors other than the presence of an SBHC (eg, school policies and infectious disease outbreaks); however, these data were not available for analysis. There is the potential for selection bias (ie, that SBHCs may be more likely to be sited in schools that already have lower absenteeism rates). To address this, we accounted for community wealth and economic disadvantage in our regression models. Our analyses did not distinguish between users and nonusers of SBHCs within the SBHC schools, although enrollment in the SBHCs is high. All data were collected before the COVID-19 pandemic; thus, our results are not impacted by any pandemic effects, although they may not be generalizable to school attendance in 2024 to 2025.

## Conclusions

This study provides evidence that rural SBHCs are associated with lower absenteeism, particularly among elementary school students. By providing primary care services at school, SBHCs may decrease absenteeism among students in rural communities. Future research should examine more recent attendance data reflecting attendance patterns during and after COVID-19–era shutdowns.

## Notes

Supplement.
Data Sharing Statement
