# Supplementary material for: School-Based Health Centers and School Attendance in Rural Areas
Source: JAMA Netw Open. 2025 May 13;8(5):e2510083. doi: 10.1001/jamanetworkopen.2025.10083 (PMC12076170; doi:10.1001/jamanetworkopen.2025.10083)
Supplement: Supplement. — Data Sharing Statement [file jamanetwopen-e2510083-s001.pdf]

Data Sharing Statement

Kjolhede. School-Based Health Centers and School Attendance in Rural Areas. *JAMA Netw Open*. Published May 13, 2025. doi:10.1001/jamanetworkopen.2025.10083

Data
Data available: No

Additional Information
Explanation for why data not available: Individual-level attendance data will not be made available to external researchers per the terms of the data use agreement with the New York State regional education service center that provided the data. The district-level data are publicly available from NYS at http://Data.NYSED.gov.
